# Author’s reply

**Published:** 2010

**Authors:** Awanish Pandey, Poonam Tripathi, R D Pandey

**Affiliations:** *Division of Pharmaceutical Sciences, I.T.M. GIDA Gorakhpur, India. E-mail: awanishpandey@rediffmail.com*

Sir,

We went through the reader’s comments[1] sent by you for reply. The comments were related to our article published in one of the previous issues of Lung India.[2] Point wise clarification is as follows:

1. Our study was based on the prescription obtained from six physicians and three different hospitals only. The study was limited to a small urban region of Gorakhpur and prescriptions obtained from hospitals were indicating toward the prevalence of asthma in males. However, study performed by Jindal *et al*.[3] was a large scale hard core epidemiological study extended up to four different major cities of India which is not comparable to limited area, prescription- based study performed by us.
2. We have taken prescription from both types of hospitals government as well as private. So chances are very less for our results to be biased from financial constraint.
